# Antimicrobial resistance of H. pylori to the outcome of 10-days vs. 7-days Moxifloxacin based therapy for the eradication: a randomized controlled trial

**DOI:** 10.1186/1476-0711-9-13

**Published:** 2010-04-15

**Authors:** Josip Bago, Karolina Majstorović, Željka Belošić-Halle, Nastja Kućišec, Vinko Bakula, Monika Tomić, Petra Bago, Rosana Troskot

**Affiliations:** 1 Department of Gastroenterology, Internal Medicine Clinic, Clinical Hospital Sveti Duh, Zagreb, Croatia; 2 Department of Microbiology, Clinical Hospital Sveti Duh, Zagreb, Croatia; 3 Department of Gastroenterology, Internal Medicine Clinic, University Hospital Mostar, Mostar, Bosnia and Herzegovina

## Abstract

**Introduction:**

Antibiotic resistance decreases success of Helicobacter pylori (Hp) eradication. Recently published results show low rate of resistance and better compliance with moxifloxacin based regiments.

**Aims&methods:**

Whether 7 days moxifloxacin with lansoprasole and amoxycillin can be compared with 10 days moxifloxacin with lansoprasole and amoxycillin according to moxifloxacin resistance. Patients with non-ulcer dyspepsia who had culture and histology positive Hp infection (n = 150) were randomly assigned into two groups. The first group (n = 75) received moxifloxacin 400 mg/d during 7 days and the other (n = 75) received moxifloxacin 400 mg/d during 10 days. All patients received amoxycillin 1 g twice daily, lansoprasole 30 mg twice daily. All Hp cultures were tested for sensitivity to moxifloxacin.

**Results:**

138 patients (92%) completed the study, 68 in the first group and 70 in the second. Eradication rates were 84% (57/68) and 76% (57/75) in the 7 days moxifloxacin group and 90% and 84% in the second group (63/70, 63/75) according to the PP and ITT analysis; p = n.s. Among 129 patients (86% of study group), 6% of strains were primary resistant to moxifloxacin.

Eradication of moxifloxacin sensitive/resistant strains was 98%/66%, p < 0.05

**Conclusion:**

According to our results we recommend 7 days moxiflixacin based triple therapy.

## Introduction

During the last few decades a large number of surveys have been conducted to show the importance of Helicobacter pylori infection as the main etiologic factor in chronic active gastritis, peptic ulcers and gastric malignancies [1]. European guidelines for Helicobacter pylori eradication recommend a triple regimen, combining proton pump inhibitor (PPI), claritromycin, and either amoxicillin or metronidazole as the first-line treatment [2,3]. Resistance to antibiotics is a major cause of eradication failure [4-6]. Currently available first-line anti H. pylori therapies may fail in up to 30% of patients [7,8] leading to significant increase of antimicrobial resistance, particularly against key antibiotics, claritromycin and/or metronidazole [9,10]. Resistance to amoxicillin and tetracycline is generally very low, even absent and is clinically insignificant [11]. To avoid resorting to rescue therapies, recent clinical trials have demonstrated that fluoroquinolone-based triple regimens may provide important options for the treatment [12-14]. Moxifloxacin-based triple regimens have been shown as a promising alternative by proving to have a good efficacy in the treatment of H. pylori infection [14-16]

Moxifloxacin is a second-generation fluoroquinolone widely used to treat respiratory and skin infections [17]. It is rapidly absorbed after oral administration, penetrates tissues well, and its half-life of 9-16 hours allows a single daily dose. The drug is well tolerated with only mild gastrointestinal disturbances (nausea and diarrhoea) as the most common side-effects [18].

According to these findings, in 2007 our group already investigated and proved the efficacy of one week of moxifloxacin-based treatment comparing with the standard treatment therapy [19].

The aim of this study was to determine the impact of moxifloxacine resistance and period of treatment on the effectiveness of a treatment regimen for eradication of H. pylori infection.

### Petients, matherials and methods

The study was conducted at the Department of Hepatogastroenterology, Internal Medical Clinic in the Clinical Hospital "Sveti Duh", Zagreb, Croatia between June 2007 and June 2008.

This was an open, randomized, comparative clinical trial with parallel groups of patients.

Before entering the study, each patient reviewed and signed an informed consent form approved by the Local Ethics Committee. Standards of Good Clinical Practice and The declaration of Helsinki were followed.

### Patients' recruitment

Consecutive patients of either sex and at least 18 years of age were recruited from those referred to Endoscopy Services for diagnostic upper-gastrointestinal endoscopy for evaluation of dyspeptic symptoms, and found to suffer from non-ulcer dyspepsia and concomitant H. pylori infection.

Patients were not suitable for the study if one of the criteria was met: diagnostically duodenal or gastric ulcers, gastrointestinal bleeding, previous attempts to eradicate H. pylori and usage of antibiotics and/or anti-gastric acid therapy within the previous 30 days and/or bismuth therapy within 90 days and usage of non-steroidal anti-inflammatory drugs, corticosteroids or gold-based drugs. The presence of severe concurrent diseases (cardio respiratory, renal, hepatic, neurological, pulmonary, metabolic, hematological and endocrine and suspected or confirmed malignancy), a condition associated with poor compliance (alcohol and drug abuse) pregnancy and breastfeeding were also exclusion criteria.

### Procedures used to diagnose Helicobacter pylori infection

Endoscopy was performed with an OlympusR FB20 endoscope (Olympus Optical Co., Tokyo, Japan) before treatment and 4-6 weeks after the treatment. Patients fasted a night before testing. Eight gastric mucosal biopsies per patient were taken with biopsy forceps (Olympus FB 24-K) in order to perform rapid urease testing (CLO test), histology and culture.

One sample each from antrum and corpus were used for the CLO test (Delta West Ltd. Bently Western Australia) which was considered positive if there was change in color within 24 hours. Histology was performed on two biopsies, each from the antrum and corpus, following the guidelines of the Sydney System. (*2004- 12-Price AB 1991*)

The biopsies were placed in separate tubes containing buffered neutral 3.7% formaldehyde solution. Hematoxylineosin stain was used to grade gastritis and Giemsa stain to detect H. Pylori.

One antral and one corpus gastric biopsy samples were taken for culture and put in a sterile receptacle containing before transporting to the microbiology laboratory within two hours after sterile pulverization specimens were placed on to fresh Columbia agar with added 7% horse blood and Iso Vitalex (BBL, Bection-Dickinson, Maryland, USA) and incubated for 3-5 days at 35-37°C under microaerophilic conditions. H. Pylori was identified as gram negative, spiral or curved rods, producing urease, catalase and oxidase.

Condition to enroll the patients in to the study was the following: two of the three tests must be positive. The treatment was considered successful if all biopsy based tests did not show the presence of H. Pylori on post-treatment assessment.

If one test suggested persistence of H. pylori a C- urea test was performed.

The ^13^C-urea breath test was performed after overnight fast and collection of baseline exhaled-breath sample. To delay gastric empting, each patient got a glass of orange juice (200 ml). Then 75 mg of ^13^C-urea dissolved in 30 ml of water was administered orally. 30 minutes later, the second breath sample was obtained. All breath samples were analyzed in an isotope-ratio mass spectrometer (IRMS, Wagner Analysen Technik, Bremen, Germany). An increase of ^13^C0_2 _exceeding the baseline value by more than 4‰ indicated the presence of H. pylori.

### Determination of H. pylori resistance

All positive cultures were tested for sensitivity to moxifloxacin and amoxicillin using E-test (AB Biodisk, Sweden), which determined minimum inhibitory concentration (MIC). The grown H. pylori colonies were resuspended in 2 li Dulbecco's modified Eagle's cell culture medium after three to five days. From this suspension 100 microliters were flooded on Columbia agar plates containing 7% horse blood. When the surface had dried, E-stripes were placed on the agar. All plates were incubated under microaerophilic conditions at 37°C as described above. They were red after 48 hours.

The MIC was defined as the lowest concentration including complete growth inhibition. The cut-off points for determining a strain as resistant were as follows: 2 μg/ml for moxifloxacin and 2.25 μml for amoxicillin.

### Treatment groups

The patients were distributed randomly to one of the following regimes:

1. LAM 7: lansoprazole 30 mg + amoxicillin 1000 mg + moxifloxacin 400 mg for 7 days

2. LAM 10: lansoprazole 30 mg + amoxicillin 1000 mg + moxifloxacin 400 mg for 10 days

A physical examination was carried out and medical history was taken during the pre-study visit. All three drugs were distributed in packs in 14 single doses and given to patients who were instructed to take drugs together two times a day, after breakfast and dinner. Each patient was asked to monitor and document all symptoms during therapy. Therapy compliance was assessed by pill count at the end of the treatment and defined as intake of >80% of the distributed medication.

Between 4 and 6 weeks after the treatment the follow up was performed, during which all changes in clinical symptoms and signs were documented. At the same time the second endoscopy was arranged, before which the investigators were not familiar with the results of E-test. While assessing the biopsy samples, the microbiologist and the pathologist were blinded to the treatment regimes, distributed to patients.

### Statistics

Analysis of efficacy of H. pylori eradication was performed on an intention-to-treat (ITT) and per protocol (PP) basis. The ITT analysis included all enrolled patients who received at least one dose of included drugs. The PP analysis included only those patients who complied with the study protocol (>80% of the assigned medication) and underwent a second endoscopy between 4 and 6 weeks after the treatment. All results of the treatment are expressed as percentages with 95% confidence intervals. The rates of H. pylori eradication in different groups were analyzed by Student's t-test. Statistical significance was taken at p < 0.05. χ^2 ^test was used to compare categorical variables, while the analysis of variance was used to compare the demographic data.

## Results

### Clinical and demographic variables, compliance and loss from follow up

Between June 2007 and June 2008 a total of 150 patients enrolled in the study. The groups did not differ in baseline characteristics, as summered in Table 1.

**Table 1:** Baseline demographic characteristics of enrolled patients

	LAM 7	LAM 10
Included in ITT analysis (n)	75	75
Age (mean ± SD)	50 ± 13	53 ± 13
Male/female (n)	35/40	37/38 p = 0.869
Smokers (n)	25	23 p = 0.8602

ITT intention-to-treat; SD standard deviation; LAM 7 lansoprazole + amoxicillin + moxifloxacin for 7 days; LAM 10: lansoprazole + amoxicillin + moxifloxacin for 10 days.

The mean age was 52 ± 13. According to gender, 48% were male and 52% were female.

All the patients were included in the ITT analysis. 12 patients (8%) did not complete the study for one of the following reasons: loss from follow up (n = 1), refusal to go under a second endoscopy (n = 3), adverse effects leading to discontinuation of treatment (n = 5), concurred disapproved medication (n = 2) and taking less than 80% of the prescribed medicines (n = 1). Those patients were excluded from PP analysis.

### Helicobacter pylori eradication

According to ITT and PP analysis with their respective 95% confidence intervals, H. pylori eradication rates did not show a significant difference between LAM 7 and LAM 10 protocols, as is presented in Table 2.

**Table 2:** H. pylori eradication rates for each treatment regimen

Protocol	ITT	PP
LAM 7	57/75 = 76%	57/68 = 84%
	(95%CI = 0.65 - 0.84)	(95%CI = 0.73 - 0.91)
LAM 10	63/75 = 84%	63/70 = 90%
	(95%CI = 0.74 - 0.91)	(95%CI = 0.81 - 0.95)
p	0.3075	0.4099

ITT intention-to-treat; SD standard deviation; LAM 7 lansoprazole + amoxicillin + moxifloxacin for 7 days; LAM 10: lansoprazole + amoxicillin + moxifloxacin for 10 days.

### Primary resistance

MIC determination was successful in samples from 92% (138/150).

Death of the H. pylori strain, fungal overgrowth or negative culture prevented MIC determination with the E-test in samples from 8% (12/150 patients). Among 129 patients (86% of the entire study group), 6% of strains were primary resistant to moxifloxacin. Eradication of moxifloxacin sensitive/resistant strains were 98%/66%, p < 0.005. All 138 strains were sensitive to amoxicillin.

### Impact of primary resistance on eradication of H. pylori

Results of our study suggest that the impact of H. pylori resistence to moxifloxacin on eradication is statisticaly significant. There was statistic significance between resistant strains and sensitive strains in LAM 7 protocol (p = 0,006), as well as in LAM 10 group (p = 0.0001).

There was no significant difference between sensitive strains in LAM 7 and sensitive strains in LAM 10 protocol.

Longer period of treatment did not provide significantly higher eradication rate, concerning both, sensitive and resistant strains.

Impact of primary resistance on eradication of H. pylori is shown in Table 3.

**Table 3:** Effect of primary moxifloxacin resistance on eradication rates

Protocol	C-s	C-r	Susceptibility unknown
LAM 7	56/63	1/4 p = 0,006	0/1
LAM 10	61/64	1/4 p = 0,0001	1/2
p	p = 0.310	p = 0.414	p = 0.665

L lansoprazole; A amoxicillin; M moxifloxacin; s sensitive; r resistant

### Adverse effects and compliance

Most adverse effects were mild to moderate in intensity. Two patients in the LAM 7 group discontinued the treatment because of diarrhoea. One patient in the LAM 10 group discontinued the treatment because of epigasrtic discomfort and two because of diarrhoea. In conclusion, side effects were more frequent with the larger period of drug usage, although the difference was not significant.

Detailed overview of adverse effects is shown in a Table 4 below.

**Table 4:** Adverse effects

	LAM 7	LAM 10
Epigastric discomfort	3	4
Nausea/vomiting	2	3
Diarrhoea	3	5
Constipation	1	0
Metallic taste	0	2
Headache	1	2
Pruritus	0	2
Skin rash	1	0
Total	11 (14.7%)	18 (0.24%) p = 0.215

LAM 7 lansoprazole + amoxicillin + moxifloxacin for 7 days; LAM 10: lansoprazole + amoxicillin + moxifloxacin for 10 days.

## Discussion

Until now, many different antimicrobial agents have been investigated, but choosing the appropriate first-line treatment for the H. pylori treatment remains a challenge.

Apart from antibiotic resistance [4-6], another reason for treatment failure lies in the fact that side-effects, longer period of treatment and amount of pills administrated per day have a negative effect on patients' compliance [20].

For those reasons, it was necessary to develop a new therapy scheme, which will result in higher eradication rates and will include fewer types of antibiotics in therapy with minor dosages and shorter period of treatment which provides better patients' compliance, fewer side-effects and lower expenses of treatment.

In the last few years, new treatment schemes based on fluoroquinolones, such as levofloxacin and moxifloxacin have been investigated all over the world.

Up to now, only few studies have evaluated a combination of levofloxacin, amoxicillin and PPI as first- line regimen. Gisbert end al. Reported in 2007 that 10-day levofloxacin- based combination represents an alternative to clarythromycin [21].

Antos and al. suggested levofloxacin tripple therapy as an option with metronidazole and clarithromycin resistant H. pylori strains [22].

However, recent studies from France, Belgium and Germany suggest that resistance of H. pylori to quinolones has already risen up to 17%, most likely due to widespread use of quinolones in other indications [23-25].

According to Italian investigators, primary resistance to levofloxacin rised up to 30.3% [26], while in Japan it amounts 15%. Indonesian scientists proved that flouroquinolones show lower resistance rates, 6.9% for ciprofloxacin, norfloxacin and ofloxacin, 2.8% for sparfloxacin and gatifloxacin, and 1.4% for levofloxacin and moxifloxacin [27].

In Southern Taiwan resistance to ciprofloxacin and levofloxacin in H. pylori collected from 2004 to 2007 increased significantly compared with the level observed during 1998 to 2003 (2.8% to 11.8%) [28].

Guided by these facts, in 2007, our group assumed and proved that moxifloxacin based treatment is effective and safe option comparing to standard triple regimens due to increased prevalence of claritromycin resistance (10.8%) [16] and metronidazole resistance (33%) [29]. Therefore, it was necessary to invent and to verify a new treatment scheme in our area, which will provide acceptable therapy outcome.

In order to improve the treatment, according to all these findings, our study group conducted this survey to determine an impact of moxifloxacine resistance and therapy duration on the effectiveness of a treatment regimen for eradication of H. pylori infection. Therefore, we compared H. pylori eradication rates, adverse effects and patients' compliance.

The eradication rates of 10-days treatment were higher than those of 7-days treatment by both ITT and PP analyses.

Adverse effects and treatment discontinuation due to adverse effects were more frequent with the larger period of drug usage, although, the difference was not significant.

According to our results we recommend 7 days moxiflixacin based triple therapy because we found no statisticaly significant eradication rates comparing LAM 7 and LAM 10 protocols.

Our assumption is that a low prevalence of fluoroquinolone resistance in Croatia may be associated with the decreased availability and the short time usage of fluoroquinolones in our practice.

In addition, it is essential to investigate the local prevalence of H. pylori resistance to antibiotics before introducing moxifloxacin in practice and, in the future, to detect a possible increase of resistance to moxifloxacin after certain period of drug usage.

## Competing interests

This study was funded in part by the Ministry of Science, Tehnology and Sports of the Republic of Croatia.

I am an employee of General Hospital Sveti Duh.

I and my authors do not have any conflict of interest.

Prim.dr.sc. J. Bago, dr.med.

## Authors' contributions

All authors have made substantial contributions to conception and design, acquisition of data, analysis and interpretation of data.
